# MCPIP1 regulates the sensitivity of pancreatic beta-cells to cytokine toxicity

**DOI:** 10.1038/s41419-018-1268-4

**Published:** 2019-01-10

**Authors:** Karolina Tyka, Anne Jörns, Jean-Valery Turatsinze, Decio L. Eizirik, Sigurd Lenzen, Ewa Gurgul-Convey

**Affiliations:** 1 Institute of Clinical Biochemistry, Hannover Medical School, 30625 Hannover, Germany; 2 ULB Center for Diabetes Research, Medical Faculty, Université Libre de Bruxelles (ULB), Brussels, Belgium; 3 Institute of Experimental Diabetes Research, Hannover Medical School, Hannover, Germany

## Abstract

The autoimmune-mediated beta-cell death in type 1 diabetes (T1DM) is associated with local inflammation (insulitis). We examined the role of MCPIP1 (monocyte chemotactic protein–induced protein 1), a novel cytokine-induced antiinflammatory protein, in this process. Basal MCPIP1 expression was lower in rat vs. human islets and beta-cells. Proinflammatory cytokines stimulated MCPIP1 expression in rat and human islets and in insulin-secreting cells. Moderate overexpression of MCPIP1 protected insulin-secreting INS1E cells against cytokine toxicity by a mechanism dependent on the presence of the PIN/DUB domain in MCPIP1. It also reduced cytokine-induced *Chop* and *C/ebpβ* expression and maintained MCL-1 expression. The shRNA-mediated suppression of MCPIP1 led to the potentiation of cytokine-mediated NFκB activation and cytokine toxicity in human EndoC-βH1 beta-cells. MCPIP1 expression was very high in infiltrated beta-cells before and after diabetes manifestation in the LEW.1AR1-iddm rat model of human T1DM. The extremely high expression of MCPIP1 in clonal beta-cells was associated with a failure of the regulatory feedback-loop mechanism, ER stress induction and high cytokine toxicity. In conclusion, our data indicate that the expression level of MCPIP1 affects the susceptibility of insulin-secreting cells to cytokines and regulates the mechanism of beta-cell death in T1DM.

## Introduction

Type 1 diabetes (T1DM) is an autoimmune disease characterized by a selective death of pancreatic beta-cells, mediated by an inflammatory process in the pancreatic islets (insulitis)^1–4^. Beta-cell destruction is mediated by CD8^+^ T cell killing^5^ and by the action of proinflammatory cytokines^1,2,6,7^. Proinflammatory cytokines released by activated immune cells infiltrating the islets activate various signaling pathways in beta-cells^1,2,6,7^ and can lead to an increase in MHC class I on the surface of beta-cells^8^. The typically secreted cytokines IL-1β, TNFα and IFNγ influence transcription, translation and cause posttranscriptional and posttranslational modifications. These changes eventually lead to nitrooxidative stress and generation of proinflammatory mediators, causing mitochondrial and ER stress responses that result in beta-cell dysfunction and damage^9–15^.

MCPIP1 (monocyte chemotactic protein–induced protein 1) is a novel antiinflammatory protein, discovered in human blood monocytes stimulated with MCP-1^16^ and in human monocyte-derived macrophages stimulated in vivo with IL-1β^17^. MCPIP1-knockdown mice suffer from severe inflammation^18^. MCPIP1 possesses a PIN-like domain with RNase and deubiquitinase properties (PIN/DUB) and is able to affect mRNA decay of several targets, including transcripts for proinflammatory cytokines (IL-1β, IL-6, IL-8) and proapoptotic proteins^19–24^. Recent studies have suggested that MCPIP1 can regulate mRNA degradation by an ARE-independent manner by binding to the stem-loop structure formed in the 3’UTR region of the targeted mRNAs^21^. MCPIP1 negatively regulates cellular inflammatory responses not only through its RNAse function, but also by deubiquitination of TRAF proteins (TRAF2, TRAF3, TRAF6) and interfering with the NFκB signaling^25,26^. MCPIP1 and NFκB regulate each other’s activity via a tight regulatory feedback-loop mechanism^24^. Targeted myocardial MCPIP1 overexpression resulted in inhibition of NFκB activity and a decrease of LPS-induced proinflammatory cytokine production, iNOS expression and caspase-3 activation^27^. Thus MCPIP1 seems to be a powerful negative regulator of inflammation.

The role of MCPIP1 in cytokine-mediated toxicity to pancreatic beta-cells in T1DM is unknown. Taking into account the important role of this protein in inflammatory processes, we decided to characterize its function in cytokine-mediated beta-cell death.

## Materials and methods

### Chemicals

Biotherm *Taq* polymerase was from GeneCraft (Münster, Germany) and Phusion High-Fidelity DNA polymerase from Thermo Fisher Scientific (Braunschweig, Germany). Cytokines were obtained from PromoCell (Heidelberg, Germany). Membranes and the ECL detection system were from Amersham Biosciences (Freiburg, Germany) and Milipore (Bedford, MA, USA). Other reagents were from Sigma Chemicals (München, Germany).

### Animal and tissues

Pancreatic islets and other tissues were obtained from 250–300 g adult male Lewis rats bred in the Central Animal Facility of Hannover Medical School according to the principles of laboratory care approved by the Local Institutional Animal Care and Research Advisory Committee of Hannover Medical School and the Lower Saxony State Office (AZ: 2014/56). Islets were isolated by collagenase digestion, separated by Ficoll gradient, and hand-picked under a stereomicroscope. Pancreatic sections were obtained from healthy and diabetic LEW.1AR1-iddm rats^28^.

### Cell culture, cytokine incubation, qRT-PCR, and RNA sequencing

INS1E cells (a kind gift of Prof.C.Wollheim, Geneva) and human EndoC-βH1 beta-cells (ENDOCELLS SARL, Paris, France;^29^) were cultured in a humidified atmosphere at 37 °C and 5% CO_2_^11,13,30^. IL-1β was used at 600 U/ml. The cytokine mixture comprised IL-1β (60 U/ml), TNFα (185 U/ml), and IFNγ (14 U/ml). Double concentrations were used with human EndoC-βH1 beta-cells, as these cells are less sensitive to cytokine-mediated toxicity^13^. The incubation time for cytokine toxicity analysis for rat INS1E cells was 24, 48 or 72-h and for human EndoC-βH1 beta-cells 7 days, based on our earlier experience^4,10,11,13^ and time-dependency experiments (Fig. S1A and Fig. 7). To analyze an impact of cytokines on gene expression cells were incubated for 6, 12 or 24-h. In each series of experiments control cells and cells with modified expression of MCPIP1 were incubated with cytokines from various batches, which was responsible for various cytokine activities between different experiments.

Total RNA was isolated using peqGOLD Total RNA columns (Peqlab, Erlangen, Germany). After quality control, 1 µg of RNA was reverse transcribed using random hexamer primers. The QuantiTect SYBR Green^TM^ technology (Qiagen) was employed^13^. For absolute quantification of *MCPIP1* gene expression an external standard curve was prepared for each target sequence (*MCPIP1, β-actin, GAPDH*) by cloning the specific cDNA into the pCR 4-TOPO vector (Thermo Fisher Scientific, Darmstadt, Germany). The gene expression of *Bip*, *C/ebpβ*, *Chop*, *iNOS*, *Mcl-1* and *MnSOD* was normalized to housekeeping genes as indicated in the figure legends. Gene expression was measured by qBasePlus system (Biogazelle, Zwijnaarde, Belgium). Primer sequences are given in supplementary Table S2.

Five human islet preparations (detailed information is provided in^31^) were exposed to IL-1β (50 U/ml) + IFNγ (1000 U/ml) for 48-hrs and RNA was sequenced as described^31^. Raw data were deposited at the Gene Expression Omnibus, submission number GSE35296.

### Genetic manipulation of MCPIP1 expression

INS1E cells were stably transfected with pcDNA3 vectors containing human wild-type MCPIP1, mutant ΔPIN/DUB MCPIP1 (a kind gift from Prof. J. Jura, Krakow, Poland^23^) or with splice variants 4 and 5 of MCPIP1 (synthesized by GeneCust, Luxembourg), or empty vector (mock) using the lipofectamine transfection method. MCPIP1 is a conserved protein in its function over various species^32^. Overexpression of human MCPIP1 in rat cells results in the generation of a fully functional MCPIP1 protein, and enables an easy tracking of the overexpression efficiency vs. induction of endogenous rat MCPIP1. This is an important control tool which allowed to distinguish the efficiency of overexpression in healthy cells from the infection/stress-induced endogenous MCPIP1 expression. Positive clones were selected against G418. MCPIP1 expression was determined by qRT-PCR and Western blotting. For inducible expression of MCPIP1 in human EndoC-βH1 beta-cells, a doxycycline-dependent overexpression Tet-ON system was used at a concentration of 25 ng/ml. Lentiviral particles for the pLVX-Tet3G vector (containing the transactivator protein) and pLVX-TRE3G-huMCPIP1 vector (containing the MCPIP1 gene under the TRE3G promoter) were generated using the Lenti-XTM Tet-On® 3G system (Clontech, Saint-Germain-en-Laye, France), according to the manufacturer’s manual. For MCPIP1 suppression cells were infected with lentiviral particles containing rat or human MCPIP1 short hairpin RNA (sc-156178-SH, sc-78944-SH, Santa Cruz Biotechnology, Heidelberg, Germany). shRNA-lentivirus particles encoding a scrambled shRNA sequence were used as a negative control (shQ). Positive clones were selected using puromycin (5 µg/ml; InvivoGen, Toulouse, France) and verified by qRT-PCR and Western blotting.

### Cytokine toxicity

Cell viability was determined after a 24-h, 48-h or 72-h incubation of INS1E cells with cytokines using a microplate-based 3-(4,5-dimethylthiazol-2-yl)-2,5-diphenyltetrazolium bromide (MTT) assay at 562/650 nm^33^. Caspase-3 or −12 activation was quantified using a green caspase-3 or red caspase-12 staining kit (PromoCell, Heidelberg, Germany) according to the instruction manual, followed by data analysis by FlowJo software (Tree Star, Ashland, OR)^3,34^. In the case of human EndoC-βH1 beta-cells the MTT assay does not provide reproducible results and therefore the PI method was used as in the case of other studies using this human beta-cell line^35–37^. The percentage of dead human EndoC-βH1 beta-cells (at least 500 cells per each condition) was determined after a 15-min incubation with the DNA-binding dye propidium iodide (PI) (50 µg/ml). The proliferation rate was quantified by using the Cell Proliferation BrdU–ELISA (Roche, Mannheim, Germany)^3^.

### Western blot analyses

Cells were homogenized in ice-cold PBS containing protease inhibitor (Roche, Mannheim, Germany) using short bursts (Braun-Sonic 125 Homogenizer, Quigley-Rochester, Rochester, NY, USA). Protein content was determined by the BCA assay (Pierce). 40 μg of total protein was resolved by SDS polyacrylamide gel electrophoresis and then electroblotted onto nitrocellulose (iNOS) or PVDF membranes (MCPIP1, P-IKKα/β and MCL-1). Immunodetection was performed using specific primary antibodies followed by secondary antibody incubation (supplementary Table S3). The hybrids were visualized using the enhanced chemiluminescence detection kit and captured by the INTAS chemiluminescence detection system (Intas Science Imaging Instruments, Göttingen, Germany).

### Nitrooxidative stress, NFκB activity, and ATP measurements

Nitrooxidative stress was detected after preincubation with 10 μM dichlorodihydrofluorescein diacetate DCFDA-H2 (30 min, 37 °C) followed by incubations with the test compounds and fluorescence measurements^10^. Nitrite accumulation was determined spectrophotometrically at 562 nm by the Griess reaction^38^. The pSEAP-NFκB construct and the Phospha-LightTM System kit (Thermo Fisher Scientific) were used to estimate NFκB activation^39^. ATP content was determined using the ATPlite Detection Assay System (PerkinElmer Life Sciences, Zaventem, Belgium) and normalized to protein content^40^.

### Immunostaining

Immunofluorescence staining in INS1E cells was performed onto collagen-coated glass slides after an overnight fixation with 4% paraformaldehyde in PBS^13,34^. Images were captured and analyzed using a Cell^R^/Olympus IX81 inverted microscope system (Olympus, Hamburg, Germany). Pancreatic sections from LEW.1 AR1-iddm rats were stained with the double immunofluorescence technique^41^. The beta-cells (stained in green by Cy2) were quantified in 10 islets per animal and subsequently analyzed for a possible co-localization of MCPIP1 (stained in red by Cy3) using an Olympus microscope BX61. The antibodies used are listed in the Table S3.

### Data analysis

All data are expressed as means ± SEM. Statistical analyses were performed using the Prism analysis program as indicated in the figure legends (Graphpad, San Diego, CA).

## Results

### Expression of MCPIP1 in rat and human insulin-secreting cells

Gene expression of *Mcpip1* was analyzed in several rat tissues (supplementary Table S4). *Mcpip1* was strongly expressed in intestine, spleen, kidney, lung and liver, in contrast to skeletal muscle where its expression was very low (~3% of expression in liver) (supplementary Table S4). The *Mcpip1* expression in rat insulin-secreting INS1E cells and in rat islets, as well as in brain was in the range of one third to half of that found in liver, respectively (supplementary Table S4).

**Fig. 1:**
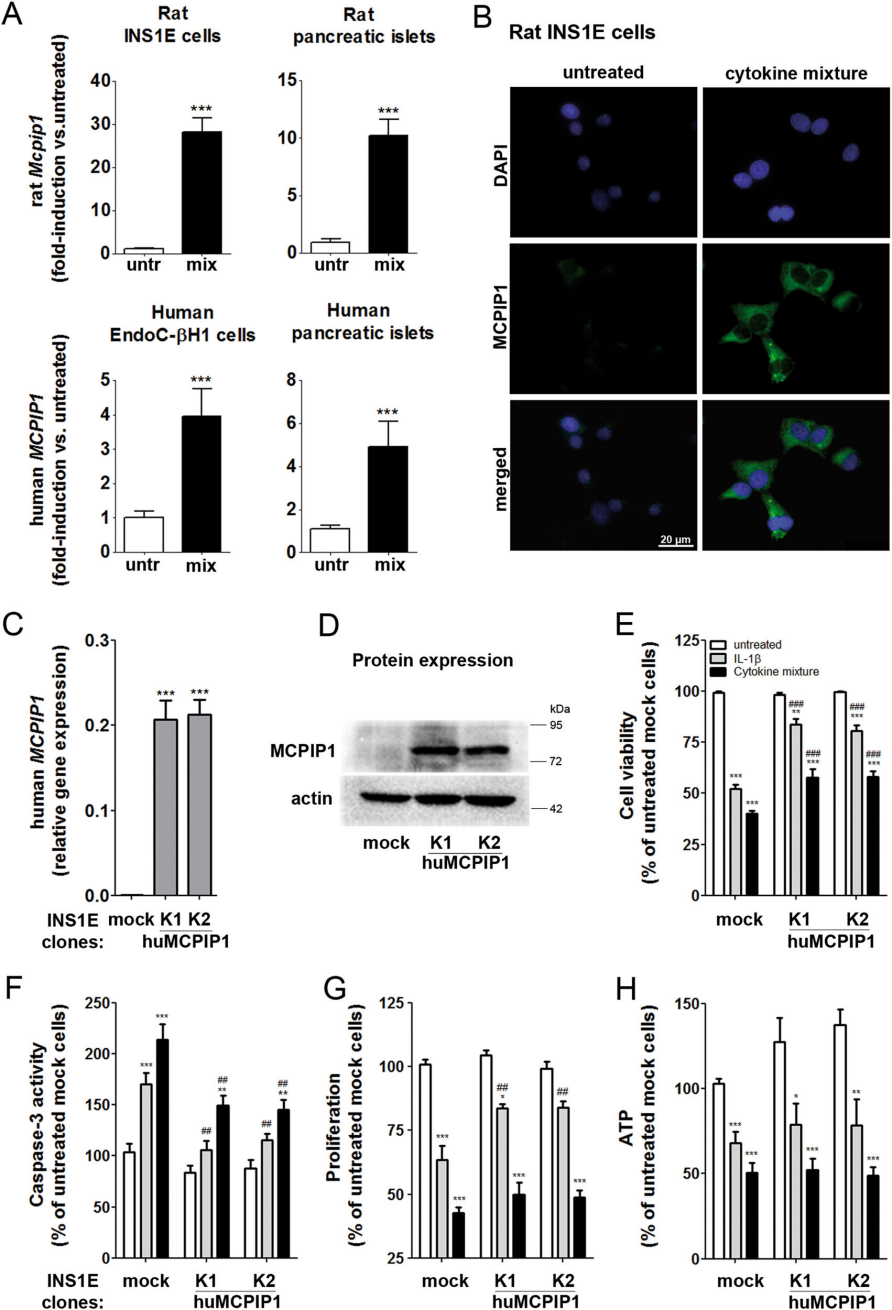
MCPIP1 expression in beta-cells and effects of MCPIP1 overexpression on cytokine-mediated toxicity in insulin-secreting INS1E cells. Real-time qRT-PCR analysis of MCPIP1 expression after 24-h incubation with a cytokine mixture (60 U/ml IL-1β, 185 U/ml TNFα, 14 U/ml IFNγ) in **a** Rat INS1E cells (relative gene expression of untreated cells: 0.023 ± 0.004, *n* = 5), rat pancreatic islets (relative gene expression of untreated cells: 0.018 ± 0.005, *n* = 4); human EndoC-βH1 beta-cells (relative gene expression of untreated cells: 2.175 ± 0.88, *n* = 5), data are means*1000 ± SEM, and human pancreatic islets (relative gene expression of untreated cells: 12.65 ± 3.89, *n* = 5, data from RNAseq^31^). **b** Immunostaining of MCPIP1 expression in untreated INS1E cells and after 24-h incubation with a cytokine mixture; green: MCPIP1, blue (DAPI): nuclei. Images were captured and analyzed using an Olympus fluorescence microscope with a 60 × oil objective. Overexpression of MCPIP1 in INS1E cells was established by stable transfection with pcDNA3-MCPIP1 vector and expression of MCPIP1 was measured by **c** qRT-PCR and **d**. Western blotting. Insulin-secreting control and MCPIP1-overexpressing INS1E cells were incubated for 24-h with IL-1β (600 U/ml) or a cytokine mixture (60 U/ml IL-1β, 185 U/ml TNFα, 14 U/ml IFNγ). **e** Cell viability was estimated by the MTT assay (*n* = 12–19); **f** Caspase-3 activation was estimated by the green caspase-3 staining kit and analyzed by FACS (*n* = 8–14); **g** Proliferation rate was analyzed by BrdU ELISA (*n* = 6–8); **h** ATP content was analyzed by ATPlite chemiluminescence assay (*n* = 5–6). Data are expressed as a percentage of the values in untreated cells. Data are means ± SEM. *p < 0.05; **p < 0.01; ***p < 0.001 vs. untreated, ^##^*p* < 0.01; ^###^*p* < 0.001 vs. control cells treated the same way, ANOVA followed by Bonferroni

The *Mcpip1* gene expression was strongly induced by a mixture of proinflammatory cytokines (60 U/ml IL-1β, 185 U/ml TNFα and 14 U/ml IFNγ) in rat INS1E cells and rat islets (Fig. 1a). The induction of MCPIP1 was observed already after a 6-h incubation with cytokines and was continuously present after longer incubation times (12 and 24-h) (Fig. S1B). In the human EndoC-βH1 beta-cells the basal expression was around 2.3-times higher than in rat insulin-secreting INS1E cells as revealed by comparison of the number of copies of *MCPIP1* normalized to the number of copies of the house-keeping gene β-actin (INS1E cells: 0.0005 vs. EndoC-βH1 beta-cells: 0.0013, *n* = 4). Furthermore, comparison of RNA sequencing data from human islets^31^ with data from primary rat beta-cells^42^ indicates a much higher expression of *MCPIP1* in human islets (20.2 ± 3.6; data shown as RPKM; *n* = 5) than in rat beta cells (0.9 ± 0.1; data shown as RPKM; *n* = 3); The cytokine-mediated increase of *MCPIP1* expression was smaller in human beta-cells than in rat beta-cells (Fig. 1a), like it was in the case of human vs. rat islets. Thus, though the basal expression of *MCPIP1* was higher in human beta-cells than in rat beta-cells, a significant increase in *MCPIP1* expression was observed in all analyzed beta-cell samples exposed to proinflammatory cytokines.

### Overexpression of MCPIP1 in INS1E cells

**Fig. 2:**
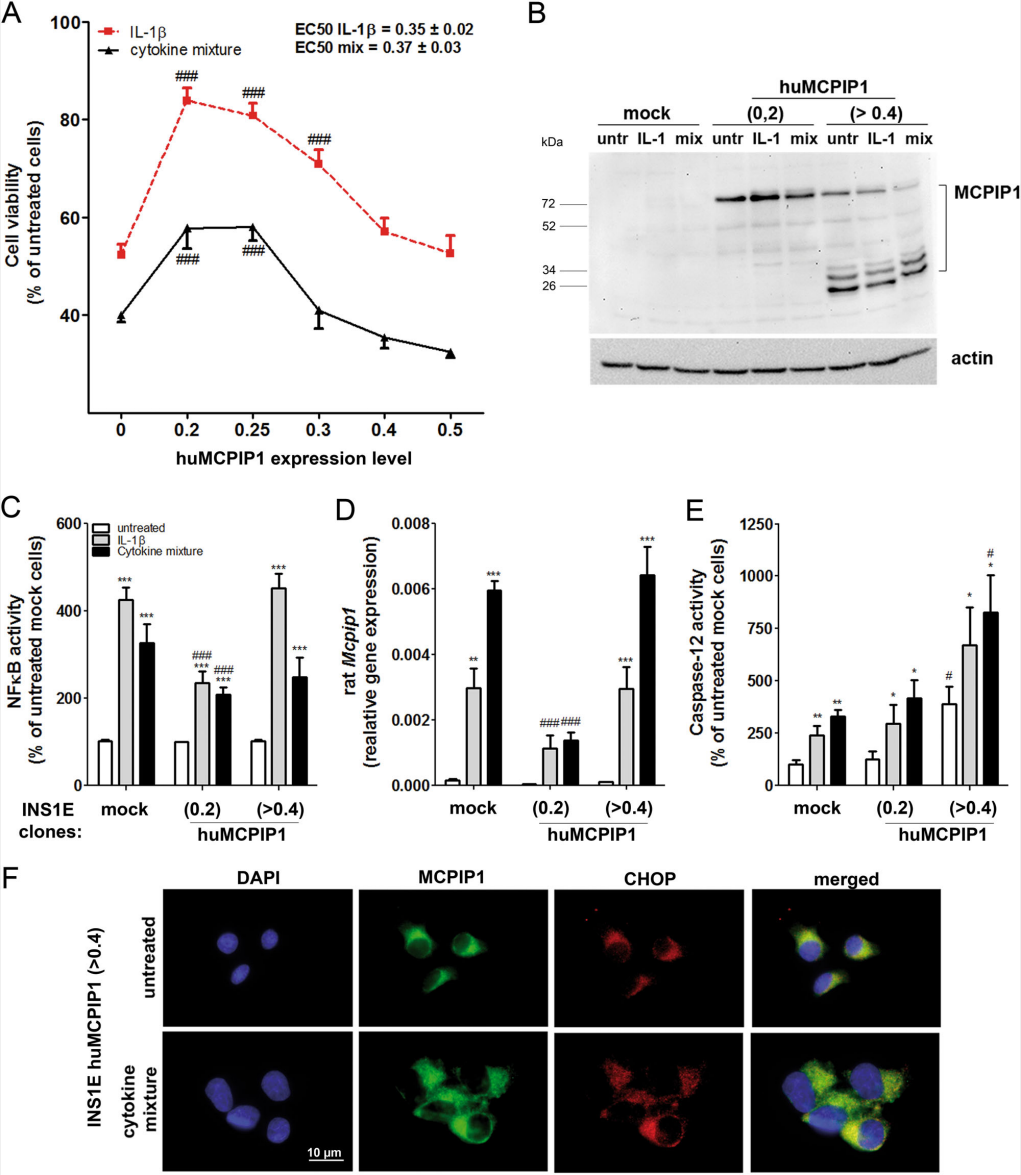
MCPIP1 overexpression in rat insulin-secreting INS1E cells. Overexpression of MCPIP1 in INS1E cells was achieved by stable transfection with the pcDNA3-MCPIP1 vector. Cells were incubated for 24-h with IL-1β (600 U/ml) or a cytokine mixture (60 U/ml IL-1β, 185 U/ml TNFα, 14 U/ml IFNγ). **a** Cell viability was estimated by MTT assay; **b** Human MCPIP1 protein expression was analyzed by Western blotting; **c** NFκB activity was measured by a SEAP-reporter gene assay; **d** Rat *Mcpip1* gene expression was analyzed by qRT-PCR and **e** Caspase-12 activation was estimated by the red caspase-12 staining kit and analyzed by FACS; **f** Representative immunostaining, green: MCPIP1, red: CHOP, blue (DAPI): nuclei. Images were captured and analyzed using an Olympus fluorescence microscope, using a 60 × oil objective. *Open bars*, untreated; *grey bars*, IL-1β; *black bars*, cytokine mixture. Data are means ± SEM of 6–8 independent experiments. *p < 0.05; **p < 0.01; ***p < 0.001 vs. untreated, ^#^*p* < 0.05; ^###^*p* < 0.001 vs. control cells treated in the same way; ANOVA followed by Bonferroni

To analyze the role of MCPIP1 in insulin-secreting cells we generated a number of cell clones either overexpressing or suppressing MCPIP1 in rat INS1E cells (Fig. 2a and S2). The expression level of overexpressed human MCPIP1 varied significantly in the cell clones and had a differential influence on the susceptibility of the cells to cytokines as measured by MTT assay. The clones with a moderate MCPIP1 overexpression were protected against cytokine toxicity, while the clones with the highest overexpression were more sensitive to cytokines (Fig. 2a). The EC_50_ value for MCPIP1 expression after which a toxic, undesirable effect was observed was 0.35 ± 0.02 (*n* = 6) for IL-1β and 0.37 ± 0.03 (*n* = 6) for the cytokine mixture. The detailed analysis of MCPIP1 protein expression in the generated clones revealed that MCPIP1 protein bands of unexpected size appeared in the clones with the highest expression level (Fig. 2b), strongly suggesting the presence of inactive misfolded protein. To check this possibility we measured cytokine-mediated NFκB activation, since NFκB is a main target of MCPIP1 activity^24^. The results revealed that cytokine-induced NFκB activation was not downregulated in the clones with the highest expression of MCPIP1 (Fig. 2c). Moreover, the feedback-loop mechanism by which MCPIP1 regulates its own transcript^24^ was also dysregulated in the clones with the highest expression of MCPIP1 (Fig. 2d). The clones with the highest expression of MCPIP1 showed clear signs of an ER overload with misfolded protein as indicated by induction of the ER stress marker CHOP expression and caspase-12 activation (Fig. 2e, f). Therefore only the clones with the overexpression below the EC_50_ value were chosen for further analysis (Fig. 1c, d). Suppression of MCPIP1 did not significantly influence the cytokine-mediated toxicity in INS1E cells (Fig. S2).

### MCPIP1 prevented the cytokine-mediated toxicity in INS1E cells

In control insulin-secreting INS1E cells (transfected with an empty vector) IL-1β and the cytokine mixture (IL-1β, TNFα and IFNγ) led to 50% loss of cell viability and strong caspase-3 activation after 24-h incubation (Fig. 1e, f). Overexpression of MCPIP1 prevented cytokine-mediated loss of cell viability and caspase-3 activation (Fig. 1e, f). The protective effect of MCPIP1 was present even in the case of a prolonged exposure to proinflammatory cytokines, however to a lesser extent as in the case of the 24-h incubation (Fig. S1A). MCPIP1 overexpression also resulted in a mild counteraction of the cytokine-induced proliferation rate decrease (Fig. 1g). The protective effect of MCPIP1 overexpression was not related to the ATP content (Fig. 1h), it seemed however to correlate with a decreased expression of MHC class I on the cell surface (Fig. S3).

### MCPIP1 inhibited the NFκB-iNOS-NO signaling pathway in INS1E cells

**Fig. 3:**
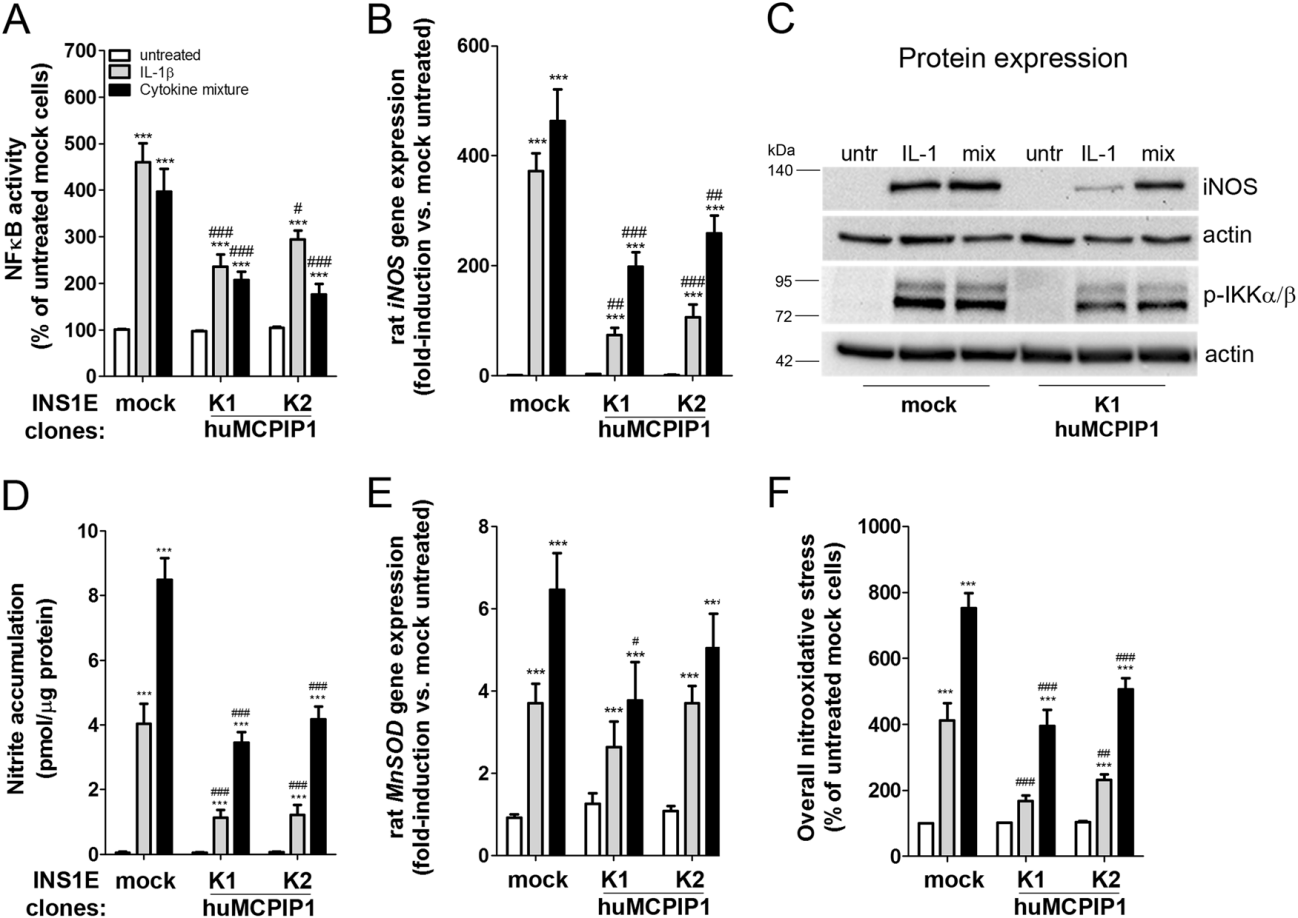
MCPIP1 regulates the activation of the NFκB-iNOS pathway in INS1E cells. Insulin-secreting control and MCPIP1-overexpressing cells were incubated for 24-h with IL-1β (600 U/ml) or a cytokine mixture (60 U/ml IL-1β, 185 U/ml TNFα, 14 U/ml IFNγ). **a** NFκB activity was measured by a SEAP-reporter gene assay (*n* = 6–10); **b** *iNOS* gene expression was analyzed by qRT-PCR (relative gene expression of untreated mock cells: 0.061 ± 0.039, mean*1000 ± SEM, *n* = 6) (*n* = 6–7); **c** Representative blots of iNOS and p-IKKα/β protein expression were obtained by Western blotting after a 24-h or 30 min incubation, respectively (*n* = 4–6); **d** Nitrite accumulation was measured by Griess assay (*n* = 8–15); **e** *MnSOD* gene expression was analyzed by qRT-PCR (relative gene expression of untreated mock cells: 18 ± 2, mean*1000 ± SEM, *n* = 8) (*n* = 6–9); **f** Overall nitrooxidative stress was analyzed by DCF fluorescence (*n* = 8–12). *Open bars*, untreated; *grey bars*, IL-1β; *black bars*, cytokine mixture. Data are means ± SEM. ***p < 0.001 vs. untreated, ^#^*p* < 0.05; ^##^*p* < 0.01; ^###^*p* < 0.001 vs. control cells treated the same way, ANOVA followed by Bonferroni

Proinflammatory cytokines stimulated the transcription factor NFκB activation in control INS1E cells (Fig. 3a). Overexpression of MCPIP1 significantly reduced cytokine-mediated NFκB activation, which correlated with decreased phosphorylation of IKKα/β (Fig. 3a, c). Consequently, the cytokine-induced *iNOS* gene expression was inhibited by MCPIP1 overexpression as compared to control INS1E cells (Fig. 3b). This went along with diminished iNOS protein expression and reduced accumulation of nitrite in cytokine-treated MCPIP1-overexpressing INS1E cells as compared to control cells (Fig. 3c, d). In contrast, the expression of *MnSOD* was not significantly downregulated by MCPIP1 overexpression (Fig. 3e). The measurement of overall nitrooxidative stress revealed a weaker increase of DCF fluorescence in the MCPIP1-overexpressing cells than in control INS1E cells (Fig. 3f).

### MCPIP1 downregulated cytokine-induced CHOP and C/EBPβ expression in INS1E cells

**Fig. 4:**
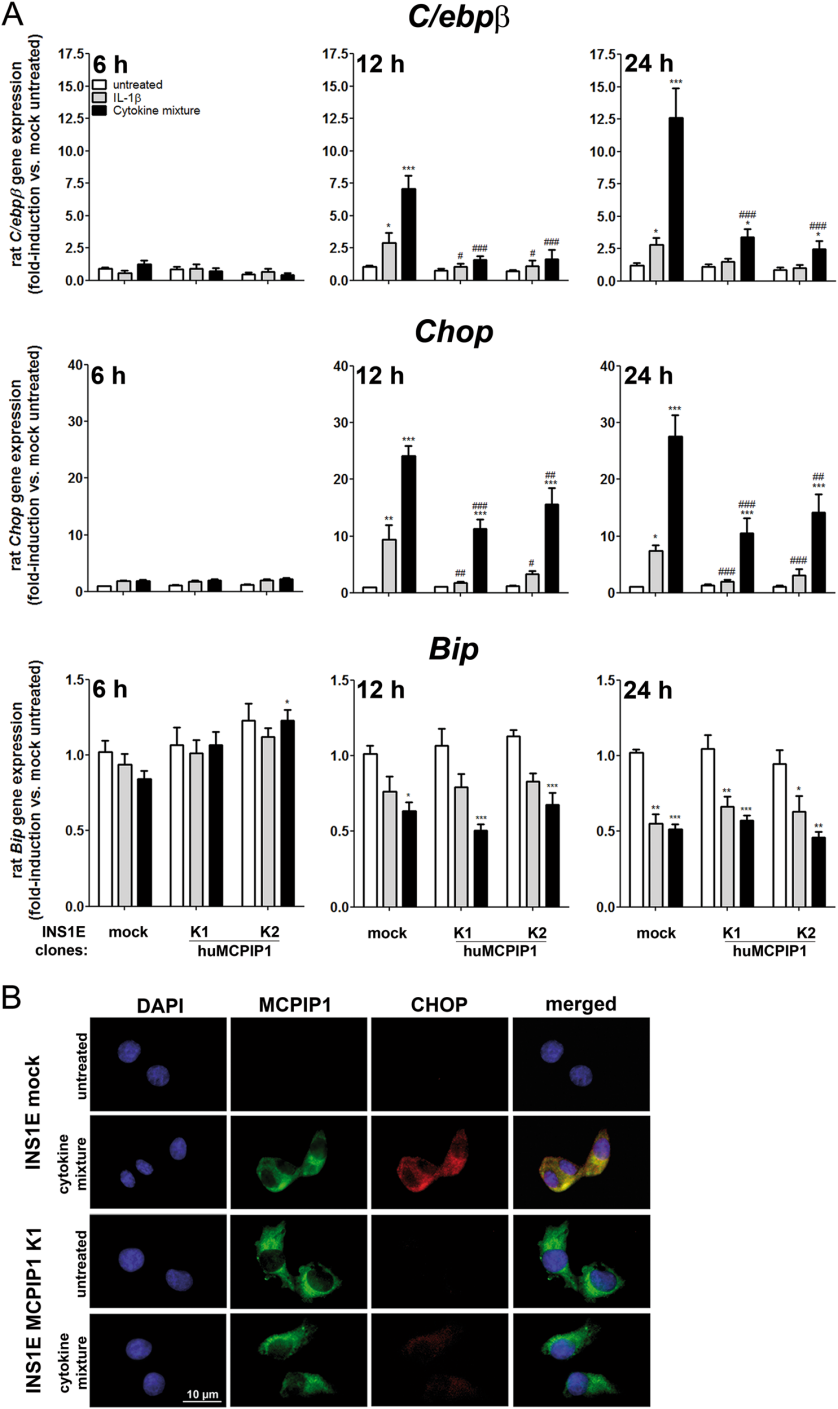
Effects of MCPIP1 overexpression on cytokine-mediated ER stress in INS1E cells. Insulin-secreting control and MCPIP1-overerexpressing cells were incubated for 6, 12 and 24-h with IL-1β (600 U/ml) or a cytokine mixture (60 U/ml IL-1β, 185 U/ml TNFα, 14 U/ml IFNγ). **a** *C/ebpβ* gene expression (relative gene expression of untreated mock cells at 24-h: 0.005 ± 0.001, mean*1000 ± SEM, *n* = 6) (*n* = 6–7); *Chop* gene expression (relative gene expression of untreated mock cells at 24-h: 6.052 ± 0.333, mean*1000 ± SEM, *n* = 8) (*n* = 6–10); *Bip* gene expression (relative gene expression of untreated mock cells at 24-h: 409 ± 44, mean*1000 ± SEM, *n* = 8) (*n* = 6–10) were measured by qRT-PCR. **b** Representative immunostaining, green: MCPIP1, red: CHOP, blue (DAPI): nuclei. Images were captured and analyzed using an Olympus fluorescence microscope, using a 60xoil objective. *Open bars*, untreated; *grey bars*, IL-1β; *black bars*, cytokine mixture. Data are means ± SEM. *p < 0.05; **p < 0.01; ***p < 0.001 vs. untreated, ^#^*p* < 0.05; ^##^*p* < 0.01; ^###^*p* < 0.001 vs. control cells treated in the same way; ANOVA followed by Bonferroni

A short time (6-h) exposure to proinflammatory cytokines did not influence the expression of *C/ebpβ*, *Chop* and *Bip* (Fig. 4a). A longer incubation (12 and 24-h) with IL-1β or a cytokine mixture led to a strong increase of *C/ebpβ* expression in control INS1E cells (Fig. 4a). In contrast, MCPIP1 overexpressing INS1E cells showed a significantly lower expression level of *C/ebpβ*, which was only mildly affected by proinflammatory cytokines (Fig. 4a). Proinflammatory cytokines induced the expression of *Chop* and slightly decreased the expression of *Bip* in insulin-secreting INS1E cells after a 12-h incubation, with a more pronounced effect observed after a 24-h incubation (Fig. 4a). Overexpression of MCPIP1 reduced cytokine-mediated CHOP induction in INS1E cells (Fig. 4a, b), but did not influence the cytokine-mediated *Bip* decrease (Fig. 4a).

### MCPIP1-mediated protection against cytokine-induced toxicity depended on the presence of the PIN/DUB domain

**Fig. 5:**
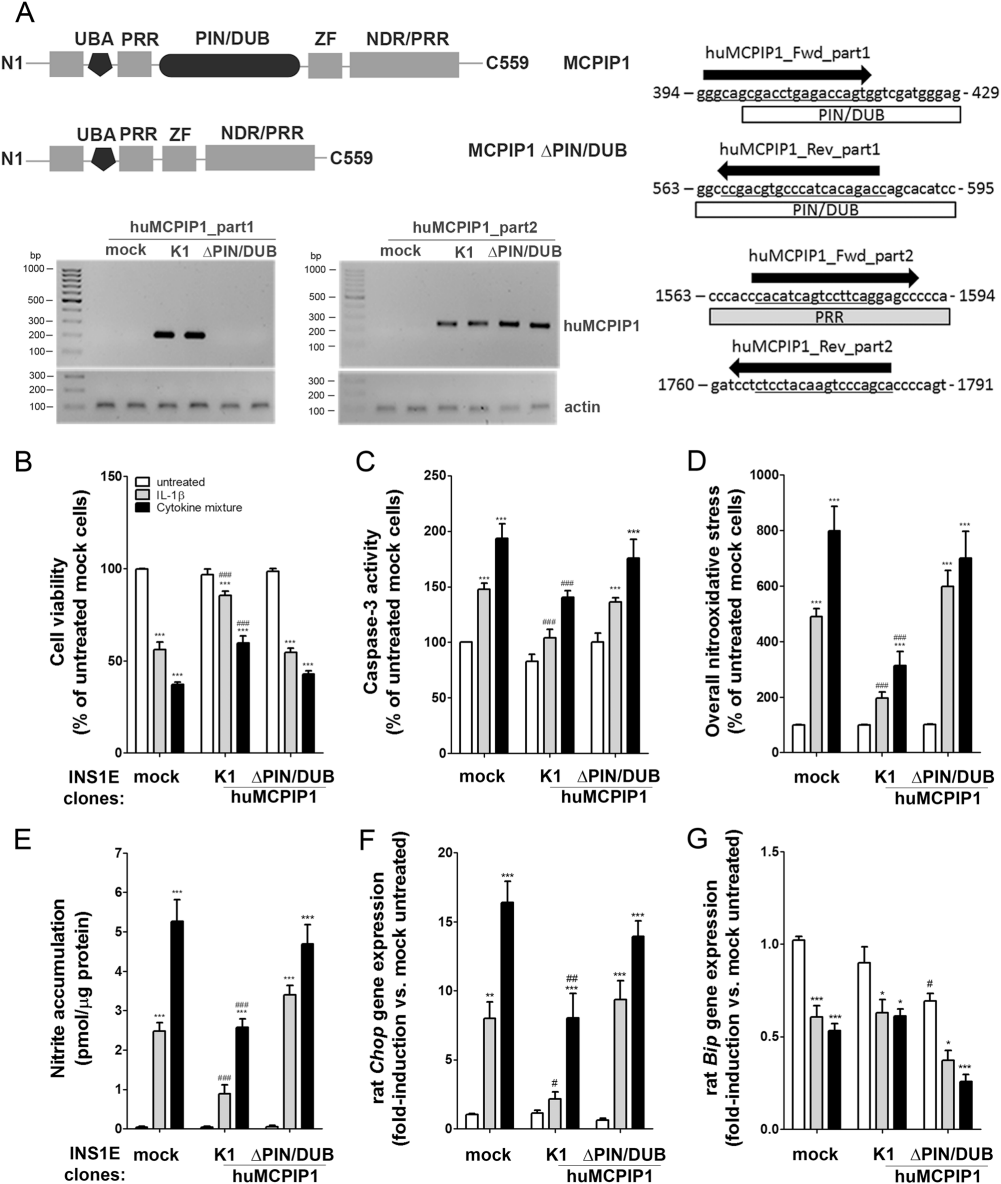
Involvement of the PIN/DUB domain of MCPIP1 in the cytokine-mediated toxicity in INS1E cells. **a** Schematic presentation of the MCPIP1 domains in the wild type and mutant ΔPIN/DUB MCPIP1 protein. DNA gel electrophoresis of qRT-PCR end-products for two MCPIP1 specific pairs of primers at two different binding sites on the MCPIP1 sequence. **b** Cell viability was measured by MTT assay (*n* = 6–8); **c** Caspase-3 activation was estimated by the green caspase-3 staining kit and analyzed by FACS (*n* = 6–9); **d** Overall nitrooxidative stress was measured by DCF fluorescence (*n* = 4–6); **e** Nitrite accumulation was analyzed by Griess assay (*n* = 4–7); **f** *Chop* gene expression was analyzed by qRT-PCR (relative gene expression of untreated mock cells: 6.052 ± 0.333, mean*1000 ± SEM, *n* = 8) (*n* = 5–8); **g** *Bip* gene expression was analyzed by qRT-PCR (relative gene expression of untreated mock cells: 409 ± 44, mean*1000 ± SEM, *n* = 8) (*n* = 5–8). INS1E insulin-secreting cells were incubated for 24-h with IL-1β (600 U/ml) or a cytokine mixture (60 U/ml IL-1β, 185 U/ml TNFα, 14 U/ml IFNγ). *Open bars*, untreated; *grey bars*, IL-1β; *black bars*, cytokine mixture Data are means ± SEM. *p < 0.05; **p < 0.01; ***p < 0.001 vs. untreated, ^#^*p* < 0.05; ^##^*p* < 0.01; ^###^*p* < 0.001 vs. control cells treated the same way; ANOVA followed by Bonferroni

In order to confirm the importance of the NFκB-iNOS-NO signaling pathway inhibition for the protection against cytokine toxicity in MCPIP1-overexpressing INS1E cells, we generated a construct with a deletion of the PIN/DUB domain and overexpressed it in INS1E cells (Fig. 5a). The successful deletion of the PIN/DUB domain was counterchecked by qRT-PCR measurements using specific primers, which enabled a distinction between native and mutant MCPIP1 forms (Fig. 5a).

The experiments showed that the PIN/DUB domain is crucial for the protective effect provided by MCPIP1 overexpression (Fig. 5). Overexpression of the mutant MCPIP1 did not counteract cytokine-mediated cell viability loss, caspase-3 activation and nitrooxidative stress (Fig. 5b–e). Also, the induction of *Chop* gene expression by cytokines was not affected by mutant MCPIP1 overexpression as compared to control cells (Fig. 5f). Overexpression of the mutant MCPIP1 decreased basal and cytokine-mediated *Bip* expression in INS1E cells (Fig. 5g).

### Overexpression of various splice variants of MCPIP1 in INS1E cells

**Fig. 6:**
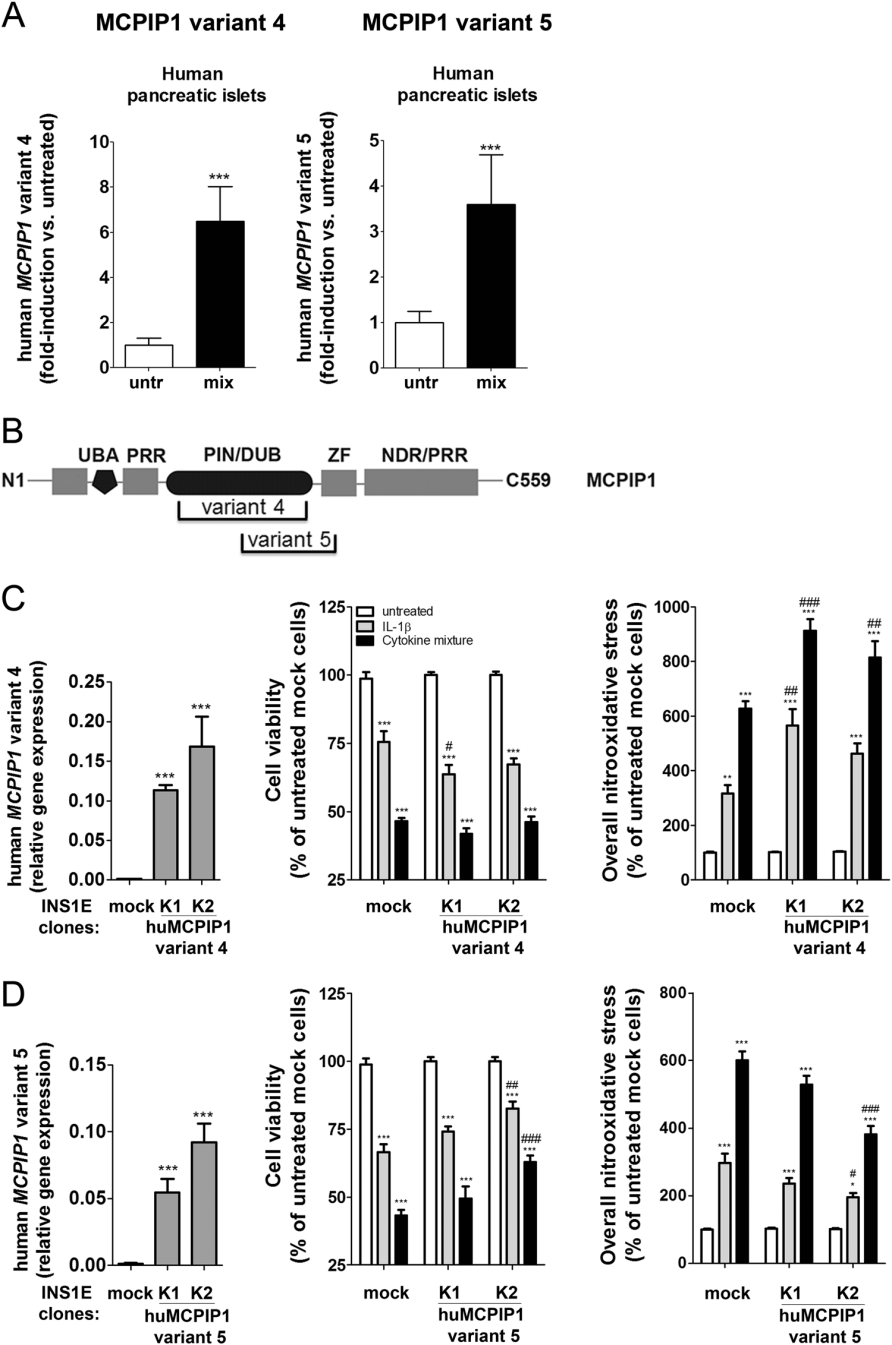
MCPIP1 splice variants in human islets and insulin-secreting INS1E cells. **a** Expression of the MCPIP1 variants 4 and 5 in human islets after exposure to a mixture of proinflammatory cytokines (IL-1β 50 U/ml and IFNγ 1000 U/ml) using RNAseq (29); **b** Scheme of the MCPIP1 splice variants 4 and 5 in comparison to the full length MCPIP1; **c** Overexpression of the MCPIP1 variant 4 in insulin-secreting INS1E cells and its effects on cytokine-mediated cell viability and induction of overall nitrooxidative stress; **d** Overexpression of the MCPIP1 variant 5 in insulin-secreting INS1E cells and its effects on cytokine-mediated cell viability and induction of overall nitrooxidative stress. Cell viability was measured by MTT assay. Nitrooxidative stress was estimated by DCF fluorescence. *Open bars*, untreated; *grey bars*, IL-1β; *black bars*, cytokine mixture. Data are means ± SEM of 4–6 independent experiments. *p < 0.05; **p < 0.01; ***p < 0.001 vs. untreated, #*p* < 0.05; ##*p* < 0.01; ###*p* < 0.001 vs. control cells treated in the same way; ANOVA followed by Bonferroni

RNA-seq analysis of human pancreatic islets exposed to the proinflammatory cytokines IL-1β + IFN-γ for 48 h, as described in^31^, revealed the presence of alternatively spliced forms of MCPIP1 (Fig. 6a). The splice variant 4 lacks the sequence coding for the PIN/DUB domain while the splice variant 5 partially lacks the PIN/DUB and ZF domains (Fig. 6b). In order to examine the function of splice variants 4 and 5, constructs containing the spliced forms were transfected into INS1E cells in which the endogenous MCPIP1 expression was suppressed. The cells were than exposed to cytokines and their viability and overall nitrooxidative stress were analyzed (Fig. 6c, d). The cytokine sensitivity of cells expressing various splice variants was done in comparison with control cells transfected with an empty vector used for splice variant expression together with silencing of the wild type of MCPIP1 (Fig. 6d). These control cells differed in their viability from the control cells transfected only with an empty vector (Fig. 1e and Fig. 6c, d). Overexpression of the splice variant 4 enhanced cytokine-mediated loss of cell viability and aggravated nitrooxidative stress (Fig. 6c). The overexpression of variant 5 led only to minor changes in response to cytokines (Fig. 6d).

### High basal expression of MCPIP1 made the human EndoC-βH1 beta-cells less susceptible to cytokine toxicity

**Fig. 7:**
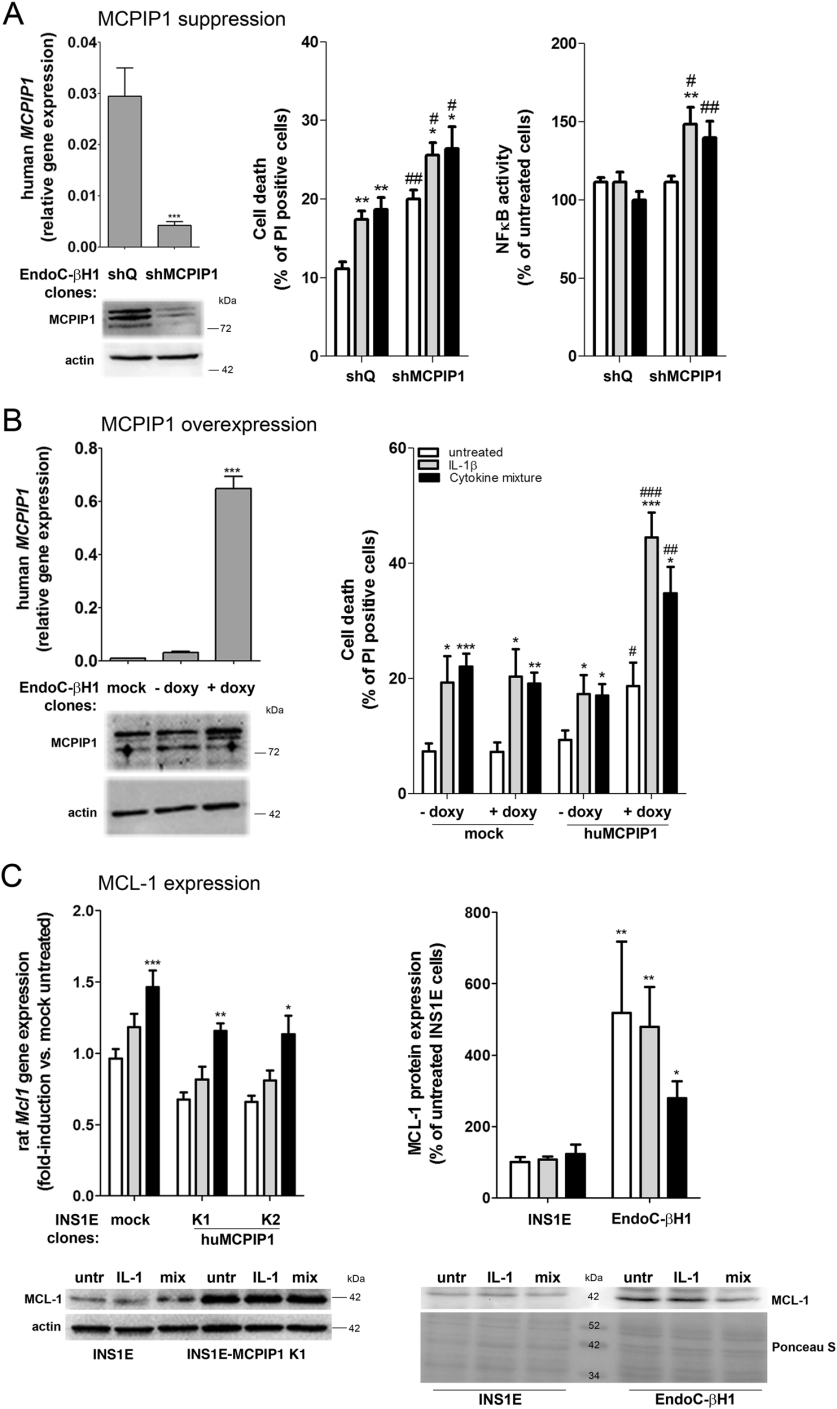
Effects of MCPIP1 on the sensitivity of human EndoC-βH1 beta-cells to proinflammatory cytokines and on the MCL-1 expression. **a** Effects of MCPIP1 suppression (by means of shRNA transfection) on cytokine-mediated cell death and NFκB activation. **b** Effects of MCPIP1 overexpression (by stable transfection with the pLVX-EF1a-Tet3-ZsGreen-MCPIP1 vector, in the presence of 25 ng/ml doxycycline). **c** Gene and protein expression of MCL-1 in rat INS1E and human EndoC-βH1 beta-cells after exposure to cytokines (24-h). Gene expression was analyzed by qRT-PCT. Protein expression was estimated by Western blotting (*n* = 4). Cytokine-mediated cell death was analyzed by PI staining, a well-established method of human beta-cell death measurement^35,36^. NFκB activation was measured by a SEAP-reporter gene assay. Human EndoC-βH1 beta-cells were incubated for 7 days with IL-1β (1200 U/ml) or a cytokine mixture (120 U/ml IL-1β, 370 U/ml TNFα, 280 U/ml IFNγ). This longer exposure time and higher concentrations of cytokines were used due to the weaker sensitivity of this cell line to cytokines^13^. *Open bars*, untreated; *grey bars*, IL-1β; *black bars*, cytokine mixture. Data are means ± SEM of 6–8 independent experiments. *p < 0.05; **p < 0.01; ***p < 0.001 vs. untreated, ^#^*p* < 0.05; ^##^*p* < 0.001; ^###^*p* < 0.001 vs. control cells treated the same way; ANOVA followed by Dunnett

The expression of MCPIP1 in untreated human EndoC-βH1 beta-cells was significantly higher than that measured in rat insulin-secreting INS1E cells (approximately 2.3-fold higher in EndoC-βH1 vs. INS1E cells). However, it was lower than in INS1E cell clones with the highest MCPIP1 expression, in which the deleterious effects of MCPIP1 were observed as described above. It is known that human beta-cells are less sensitive to proinflammatory cytokines than rat beta-cells and the cytokine toxicity develops over a longer time as compared to rat beta-cells^13^. To investigate the role of MCPIP1 in the differential susceptibility of beta-cells to cytokine toxicity we generated human EndoC-βH1 beta-cell clones overexpressing or suppressing MCPIP1 (Fig. 7a, b). We chose the PI staining for the analysis of cytokine-mediated toxicity in this cell line, since this method provides the most reproducible results and is typically used by other groups working with these cells^35–37^. The analysis of cell death of EndoC-βH1 beta-cells by PI staining revealed that shRNA-mediated suppression of MCPIP1 led to a significant 2-fold increase in cell death already in untreated cells (Fig. 7a). The cell death rate after incubation with cytokines was significantly higher after MCPIP1 suppression (Fig. 7a). Interestingly, and in contrast to control EndoC-βH1 beta-cells, there was a significant activation of NFκB after cytokine exposure in human beta-cells with a MCPIP1 knock-down (Fig. 7a). Importantly, a very mild overexpression of MCPIP1 (doxycycline doses up to 15 ng/ml) was not protective to human EndoC-βH1 cells (data not shown). Induction of MCPIP1 by a slightly higher dose of 25 ng/ml of doxycycline led to increased cell death (Fig. 7b). Thus the basal expression of MCPIP1 in untreated human EndoC-βH1 beta-cells was around twice as high as it was in untreated rat INS1E cells and this correlated with reduced NFκB activation and lower susceptibility towards proinflammatory cytokines.

### MCPIP1 maintained a high expression of the antiapoptotic MCL-1 protein

MCL-1 is one of the most important antiapoptotic proteins in beta-cells^36^. The gene expression of *Mcl1* was increased by IL-1β alone and the cytokine mixture in insulin-secreting INS1E cells (Fig. 7c), thus confirming earlier observations^43^. MCPIP1 overexpressing INS1E cells showed a slightly lower endogenous gene expression of *Mcl1* as compared to control cells (Fig. 7c). On the protein level the expression of MCL-1 was not significantly influenced in INS1E cells by 24-h incubation with 600 U/ml IL-1β or a cytokine mixture (Fig. 7c). Interestingly, the protein expression of MCL-1 was significantly higher in INS1E cells overexpressing MCPIP1 than in control INS1E cells (Fig. 7c).

Moreover, human EndoC-βH1 beta-cells were characterized by a strong and significantly higher basal expression of MCL-1 as compared to rat INS1E cells (Fig. 7c). Incubation with IL-1β alone or a mixture of proinflammatory cytokines slightly decreased the MCL-1 protein expression in EndoC-βH1 beta-cells (Fig. 7c). Control experiments with the same cytokine concentrations and combinations as by^36^ reduced significantly MCL-1 protein expression (data not shown), thus confirming earlier observations. Thus, a moderate expression of MCPIP1 correlated with a strong expression of the antiapoptotic protein MCL-1.

### MCPIP1 expression fluctuated during diabetes development in the LEW.1AR1-*iddm* rat, a model of human T1DM

**Fig. 8:**
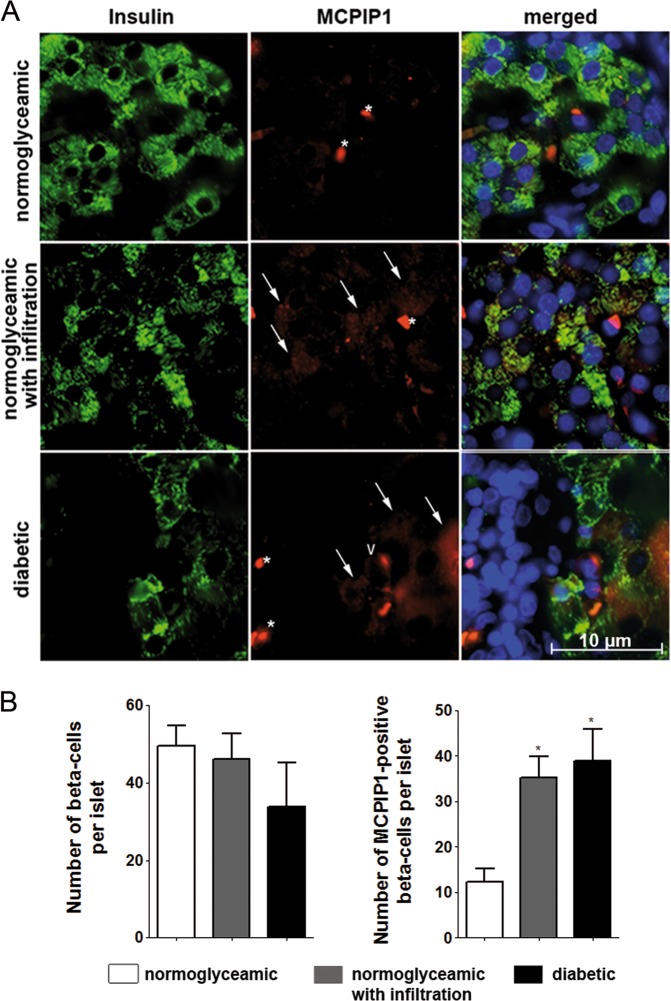
MCPIP1 expression under different metabolic conditions in the LEW.1AR1-iddm rat, a model of human type 1 diabetes mellitus. Pancreatic islets from the IDDM rat were analyzed for MCPIP1 expression under various metabolic conditions (detailed information in Table S1). **a** Representative pictures of immunostaining, green: insulin, red: MCPIP1, blue (DAPI): nuclei. Images were captured and analyzed using an Olympus fluorescence microscope, using a 60x oil objective. White asterix: erythrocyte, white arrow: MCPIP1-positive beta-cell, white V: MCPIP1-postive non-beta-cell. **b** Quantification of the absolute number of MCPIP1-positive beta-cells in pancreatic islets of IDDM rats. Four animals and ten islets per animal in each group were analyzed. Data are presented as means±SEM. *p < 0.05 vs. MCPIP1-positive beta-cells in normoglycaemic without islet infiltration

The *Mcpip1* expression was analyzed also in islets from the IDDM rat **(**LEW.1AR1-*iddm* rat), a model of human T1DM (supplementary Table S1 and Fig. 8). In each islet around 50 beta-cells were analyzed, with exception of the rats with overt diabetes, in which a concomitant loss of insulin-positive beta-cells was observed (Table S1, Fig. 8). The number of MCPIP1-positive beta-cells differed significantly in healthy vs. prediabetic IDDM rats with normoglycaemia and signs of islet infiltration as well as in diabetic IDDM rats with hyperglycaemia and severe islet infiltration (Table S1, Fig. 8). In normoglycaemic rats without any signs of islet infiltration only 12% of beta-cells were MCPIP1-positive with a relatively faint expression level (Table S1, Fig. 8). In the prediabetic phase with islet infiltration, the number of double positive cells increased significantly to 35% (Table S1, Fig. 8). In hyperglycaemic rats with severe signs of islet infiltration and beta-cell loss the number of MCPIP1-positive beta-cells was around 39%, also significantly higher than in normoglycaemic rats (Table S1; Fig. 8).

## Discussion

The novel cytokine-induced protein MCPIP1 is an interesting candidate for future antiinflammatory therapies in the context of several inflammatory disorders^19,32,44–46^. To date there has been no information about the role of MCPIP1 in pancreatic beta-cells and its significance for T1DM.

To uncover the role of MCPIP1 in beta-cells we employed genetically modified insulin-secreting cell lines of rat and human origin. Interestingly, we observed a higher basal expression of MCPIP1 in human than in rat insulin-secreting cells. This correlated with the lower susceptibility to proinflammatory cytokines and reduced cytokine-mediated MCPIP1 induction in human vs. rat beta-cells. The transcription factor NFκB plays a crucial role in cytokine-induced cell death of rat beta-cells^6,47^ by a mechanism strongly dependent on the induction of the iNOS-NO pathway and nitrooxidative stress-mediated disturbances of mitochondria and ER^3,9–15^. In contrast, proinflammatory cytokines fail to induce the iNOS pathway in human beta-cells, an effect accounted for the weaker activation of the NFκB in human beta-cells^10,13,35^. Our data indicate that this difference between human and rat beta-cells could be explained at least partially by the higher basal MCPIP1 expression in human beta-cells, reducing the activation of NFκB by a regulatory feedback-loop as described in hepatoma cells^24^.

Proinflammatory cytokines induced a rapid and strong MCPIP1 expression in insulin-secreting cells of various origins as well as in primary rat and human islets. Importantly, we confirmed these in vitro observations in a rat model of human T1DM, the IDDM rat **(**LEW.1AR1-*iddm* rat)^28^. Upon infiltration of islets, but before hyperglycaemia, the number of MCPIP1-positive beta-cells as well as the magnitude of MCPIP1 expression significantly increased and were maintained at a high level after diabetes manifestation. These data suggest that MCPIP1 might be involved in the regulation of the pancreatic beta-cell fate during T1DM development. Further analyses in pancreatic sections of T1DM patients at various onsets of the disease are however needed to confirm a role of MCPIP1 in human diabetes.

Interestingly, in rat INS1E cells the cytokine-mediated cell viability loss and caspase-3 activation were prevented by moderate overexpression of MCPIP1, but potentiated by its very strong overexpression. Suppression of MCPIP1 in INS1E cells did not affect cytokine toxicity since the basal MCPIP1 expression in rat insulin-secreting cells was already very low. The protective effect of moderate MCPIP1 overexpression in INS1E cells was strongly dependent on the inhibition of the cytokine-mediated phosphorylation of IKKα/β, reduction of NFκB activation and decrease of iNOS gene and protein expression, resulting in a reduced nitrite accumulation. In human beta-cells NO generation is not induced by cytokines^13,35^, and this correlates with a slower development of cell death. Indeed suppression of MCPIP1 resulted in a potentiation of cytokine toxicity in human EndoC-βH1 beta-cells together with a parallel increase of NFκB activation after cytokine exposure. Interestingly, a weak overexpression of MCPIP1 in human EndoC-βH1 beta-cells did not influence cytokine toxicity, while a stronger overexpression had similar effects to the high, deleterious overexpression in rat INS1E cells. This observation suggests that the basal expression of MCPIP1 in human EndoC-βH1 beta-cells, which are characterized by a weaker sensitivity to cytokine-mediated toxicity than rat INS1E cells, resembles the protective moderate overexpression of MCPIP1 in the rat beta-cell line. The toxic effects of a high overexpression of MCPIP1 in rat INS1E cells were attributed to the accumulation of misfolded MCPIP1 protein and disturbances in the ER. In contrast, moderate MCPIP1 overexpression resulted in a reduced cytokine-mediated *Chop* expression in INS1E cells, indicative of ER stress inhibition since CHOP induction is a central event in cytokine-induced ER stress^48^.

The protein C/EBPβ has been shown to be involved in ER stress activation via binding to ATF4 and CHOP^49^. Here we showed that *C/ebpβ* expression was significantly induced by proinflammatory cytokines in control-INS1E cells, while its basal expression was significantly lower and not affected by cytokines after moderate MCPIP1 overexpression. This is in line with previous findings showing a cleavage of C/EBPβ by MCPIP1^22^.

The protective effect of moderate MCPIP1 overexpression in rat INS1E cells as well as the lower susceptibility of human EndoC-βH1 beta-cells towards proinflammatory cytokines strongly correlated with the MCL-1 expression level. MCL-1 has been recently shown to be a crucial antiapoptotic protein in rat and human beta-cells and is markedly downregulated in human islets of T1DM patients^36,43^. MCL-1 turnover depends on the ubiquitination status^36^. MCPIP1 contains a PIN/DUB domain which has RNAse and deubiquitynation properties as well an UBA domain which participates in the ubiquitination regulation^32^. Transfections of mutant or spliced forms of MCPIP1 revealed that the protective effects of MCPIP1 can be attributed to the function of the PIN/DUB domain. The PIN/DUB domain was responsible for the reduction of NFκB-iNOS pathway induction as well as the prevention of ubiquitination and degradation of the key antiapoptotic protein MCL-1. We observed also a reduced expression of the ER chaperone *Bip* after overexpression of the mutant MCPIP1 lacking the PIN/DUB domain activity, as it was shown in a recent study in renal carcinoma cells^50^. Our data suggest that splice variants of MCPIP1 could be involved in the fine tuning of the MCPIP1 action in beta-cells, a process likely to be dysregulated under unresolved inflammation during T1DM development, which may lead to uncontrolled expression of MCPIP1 and increased cytokine toxicity. These results strengthen the recently expressed view that modified alternative splicing is an important mechanism by which inflammation contributes to beta-cell dysfunction and death^51^.

Our in vitro data show an antiapoptotic, antiinflammatory potential of MCPIP1 in rat beta-cells upon a moderate overexpression and a toxic effect in the case of a very strong overexpression, a situation which might occur in the case of severely infiltrated islets in the IDDM rat. Noteworthy, in hyperglycaemic IDDM rats beta-cells show a higher expression of caspase-3 and perforin in severely infiltrated islets^52^. The main difference between faintly and severely infiltrated islets, besides the number and localization of the immune cells, was the dissociated islet architecture that can lead to new interactions between beta-cells and other endocrine as well as stromal cells^41^. Changes in MCPIP1 expression in islets upon infiltration could also influence the T-cell receptor expression on the activated T-cells within the islet infiltrate, thereby changing the entire milieu as recently shown for psoriasis^44^. Fluctuations of MCPIP1 expression could also affect the macrophage polarization at the site of insulitis, as shown in other inflammatory disorders^53^ or MHC class I expression on beta-cell surface as shown in our study.

In conclusion, MCPIP1 is a novel, powerful cytokine-induced protein in beta-cells that regulates beta-cell cytokine susceptibility by affecting a number of cytokine-sensitive pathways. Since MCPIP1 expression undergoes fluctuations in islets during T1DM development in the IDDM rat and regulates the beta-cell fate upon exposure to proinflammatory cytokines, manipulations of its expression and activity might open new perspectives for T1DM treatment.

## Supplementary information

Supplemental material
