# Supplementary material for: MCPIP1 regulates the sensitivity of pancreatic beta-cells to cytokine toxicity
Source: Cell Death Dis. 2019 Jan 10;10(1):29. doi: 10.1038/s41419-018-1268-4 (PMC6328635; doi:10.1038/s41419-018-1268-4)
Supplement: Supplementary file 1 — Supplemental material [file 41419_2018_1268_MOESM1_ESM.docx]

**Supplementary Information**

**MCPIP1 regulates the sensitivity of pancreatic beta-cells to cytokine toxicity**

**Karolina Tyka^1^, Anne Jörns^1^, Jean-Valery Turatsinze^2^, Decio L. Eizirik^2^, Sigurd Lenzen^1,3^ and Ewa Gurgul-Convey^1*^**

From ^1^Institute of Clinical Biochemistry, Hannover Medical School, 30625 Hannover, Germany, ^2^ULB Center for Diabetes Research, Medical Faculty, Université Libre de Bruxelles (ULB), Brussels, Belgium, ^3^Institute of Experimental Diabetes Research, Hannover Medical School, Hannover, Germany

Running head: MCPIP1 in beta-cells

*Address correspondence and requests for reprints to: Dr. Ewa Gurgul-Convey, Institute of Clinical Biochemistry, Hannover Medical School, 30625 Hannover, Germany
Phone: + 49/511/5326780, Fax: + 49/511/5323584, Email: [Gurgul-Convey.Ewa@mh-hannover.de](mailto:Gurgul-Convey.Ewa@mh-hannover.de)

**This section contains the information about primers (Table S1) and antibodies (Table S2) used in the study as well as additional data on the MCPIP1 expression in the LEW.1AR1-iddm rat and various rat tissues (Table S1 and S4). In Fig.S1 time-dependent expression of MCPIP1 in rat INS1E cells after exposure to proinflammatory cytokines as well as effects of a prolonged exposure to proinflammatory cytokines (48-h and 72-h) on INS1E MCPIP1-overexpressing cell are shown. The effects of MCPIP1-suppression on cytokine mediated toxicity in rat INS1E cells are presented in Fig.S2. Additionally, the analysis of MHC class I expression in INS1E cells with overexpression or suppression of MCPIP1 in the absecence or presence of cytokines is shown in Fig.S3.**

**SUPPLEMENTARY TABLES**

**Supplementary Table. S1. MCPIP1 expression in beta-cells under different metabolic conditions in the LEW.1AR1-*iddm* rat, a model of human type 1 diabetes mellitus.**

| **LEW.1AR1-*iddm* rats** | Blood glucose (mmol/l) | Number of beta-cells  per islet | Number of MCPIP1-positive beta-cells per islet | % of MCPIP1-positive beta-cells per islet |
| --- | --- | --- | --- | --- |
| Normoglycaemic without islet infiltration | 5.3 ± 0.4 | 49.6 ± 5.2 | 7.1 ± 1.7 | 12.3 ± 3.0 |
| Normoglycaemic  with islet infiltration | 5.6 ± 0.3 | 46.2 ± 6.6 | 18.2 ± 3.0 * | 35.2 ± 4.7* |
| Hyperglycaemic  with islet infiltration | 16.3 ± 1.2 | 33.7 ± 11.4 | 10.1 ± 1.7 | 38.9 ± 7.0* |

The absolute number of MCPIP1-positive beta-cells in pancreatic islets in LEW.1AR1-*iddm* rats. 4 animals and 10 islets per animal in each group were analyzed. Data are presented as mean values ± SEM. **p* < 0.05 vs. MCPIP1-positive beta-cells in normoglycemic without islet infiltration.

**Supplementary Table S2. Primer sequences used for qRT-PCR.**

| **Gene** | **Forward 5’-3’** | **Reverse 5’-3’** |
| --- | --- | --- |
| **rat** **β-actin** | GAACACGGCATTGTAACCAACTGG | GGCCACACGCAGCTCATTGTA |
| **rat *Bip*** | CCACCAGGATGCAGACATTG | CGCACTGACCACTCTGTTTC |
| **rat *C/ebpβ*** | CAAGATGCGCAACCTGGAGA | AGCTGCTTGAACAAGTTCCG |
| **rat *Chop*** | CCAGCAGAGGTCACAAGCAC | CGCACTGACCACTCTGTTTC |
| **rat *Gapdh*** | AGTTCAACGGCACAGTCAAG | AAGGCCTCACTCTGTTTGCGGA |
| **rat *G6PDH*** | ATCCTACCATCTGGTGGCTGTTCC | AAGGCCTCACTCTGTTTGCGGA |
| **rat *iNOS*** | TCGTACTTGGGATGCTCCATGG | TCCTGCAGGCTCACGGTCAA |
| **rat *Mcpip1*** | TCCAAGAAATCAGCCCCACC | CCACCTTCATCTGCAGCTCG |
| **rat *Mcpip1* part 1** | GGCAGCGACCTGAGACCAGTG | GGTGTGTGATGGGCACGTCGG |
| **rat Mcpip1 part 2** | CACATCAGTCCTTCAGGAG | TGCTGGGACTTGTAGGAG |
| **rat *Mcl1*** | CCTCCAGCCACCAACTACAT | CCACTTTCTTTCTGCCGTGTT |
| **rat *MnSOD*** | CCTCCCTGACCTGCCTTACGACTA | TTCAGATTGTTCACGTAGGTCGCG |
| **human *MCPIP1*** | GGCAGCGACCTGAGACCAGTG | GGTGTGTGATGGGCACGTCGG |
| **human β-actin** | ATGGATGATGATATCGCCGC | TTCTGACCCATGCCCACCA |

**Supplementary Table S3. Antibodies used in Western blotting and immunofluorescence analyses**

| **Protein** | **Antibody** | **Company** |
| --- | --- | --- |
| **iNOS** | rabbit polyclonal IgG, M-19:sc-650,  1:300 (WB) | Santa Cruz |
| **MCPIP1** | polyclonal antibodies,  1:3,000 (WB) | Dr.Jura lab, Krakow, Poland |
| **MCPIP1**  **(in pancreatic sections)** | mouse monoclonal IgG, P-12:sc-136750,  1:100 (IF), 1:500 (WB) | Santa Cruz |
| **P-IKK** | rabbit monoclonal, No. 2694,  1:500 (WB) | Cell Signalling |
| **MCL-1** | rabbit polyclonal IgG, 3035-100,  1:500 (WB) | BioVision |
| **MHC I** | mouse monoclonal, RT1A/OX-18  1:100 (IF) | BioRad |
| **β-actin** | mouse monoclonal, C-4 sc-47778, 1:500 (WB) | Santa Cruz |
| **CHOP** | rabbit polyclonal clone D467F1 ,  1:200 (IF) | Cell Signalling |
| **insulin** | mouse monoclonal clone D3E7,  1:400 (IF) | BioRad |
| **Secondary Alexa Dylight** | 488-conjugated anti-mouse IgG,  1:400 (IF) | Dianova |
| **Secondary**  **Cyanine2, Cy3,Cy5** | anti-rabbit IgG, anti-mouse IgG  1:400 (IF) | Dianova |
| **secondary peroxidase conjugated Affini Pure IgG (H+L)** | Donkey anti-rabbit  1:40,000 (WB) | Dianova |

**Supplementary Table S4. MCPIP1 gene expression in various rat tissues.**

| **Tissue** | **Rat *Mcpip1* (%)** |
| --- | --- |
| Intestine | 337 ± 47 (6) ** |
| Spleen | 277 ± 49 (4) ** |
| Kidney | 198 ± 34 (3) ** |
| Liver | 100 ± 6 (4) * |
| Lung | 82 ± 16 (4) |
| Pancreatic islets | 45 ± 17 (6) * |
| Heart muscle | 40 ± 2 (4) ** |
| INS1E | 37 ± 11 (6) ** |
| Brain | 29 ± 4 (4) ** |
| Skeletal muscle | 3 ± 1 (4) ** |

Total RNA was isolated from different rat tissues. qRT-PCR was performed to determine rat *Mcpip1* expression. *Mcpip1* expression was normalized to *β-actin*, *Gapdh* and *G6PDH*. Data are mean values ± SEM, with the numbers of experiments provided in parentheses. The value for liver was set as a 100%.
* p<0.05, ** p<0.01; ANOVA followed by Bonferroni.

**SUPPLEMENTARY FIGURES**

**Supplementary Fig. S1. Effects of MCPIP1 on cytokine-mediated beta-cell viability in INS1E cells**

A. Insulin-secreting control and MCPIP1-overexpressing cells were incubated for 24, 48 and 72-h with IL-1β (600 U/ml) or a cytokine mixture (60 U/ml IL-1β, 185 U/ml TNFα, 14 U/ml IFNγ) and cell viability was measured by MTT assay, n=5-6. Data are expressed as a percentage of the values in untreated cells; B. Real-time qRT-PCR analysis of *Mcpip1* expression after 6, 12 and 24-h incubation with IL-1β (600 U/ml) or a cytokine mixture (60 U/ml IL-1β, 185 U/ml TNFα, 14 U/ml IFNγ), n=4-6. Data are means ± SEM. *Open bars*, untreated; *grey bars*, IL-1β; *black bars*, cytokine mixture. **p<0.01, ***p<0.001 vs. untreated, #p<0.05, ##p<0.01, ###p<0.001 vs. control cells treated the same way, ANOVA followed by Bonferroni.

**Supplementary Fig. S2. Effects of MCPIP1 suppression on cytokine-mediated beta-cell toxicity in INS1E cells**

Insulin-secreting control and MCPIP1-suppressing cells were incubated for 24-h with IL-1β (600 U/ml) or a cytokine mixture (60 U/ml IL-1β, 185 U/ml TNFα, 14 U/ml IFNγ). Expression of MCPIP1 was measured by A. qRT-PCR; B. iNOS protein expression was analyzed by Western blotting; C. Cell viability loss was estimated by the MTT assay (n=5-6); D. Overall nitrooxidative stress was analyzed by DCF (n=5-6); E. nitrite accumulation was measured by Griess assay (n=4-6). Data are expressed as a percentage of the values in untreated cells. Data are means ± SEM. *Open bars*, untreated; *grey bars*, IL-1β; *black bars*, cytokine mixture. ***p<0.001 vs. untreated, ANOVA followed by Bonferroni.

**Supplementary Fig. S3 Effects of MCPIP1 on MHC class I expression in rat insulin-secreting INS1E cells**

Insulin secreting INS1E cells with a genetically modified expression level of MCPIP1 were incubated for 24 h with IL-1β alone or a cytokine mixture. Thereafter the immunostaining for MHC class I was performed; green: MHC class I, red: insulin, blue (DAPI): nuclei. Images were captured and analyzed using an Olympus fluorescence microscope, using a 100 × oil objective.

**

**

**Supplementary Fig. S1**

**
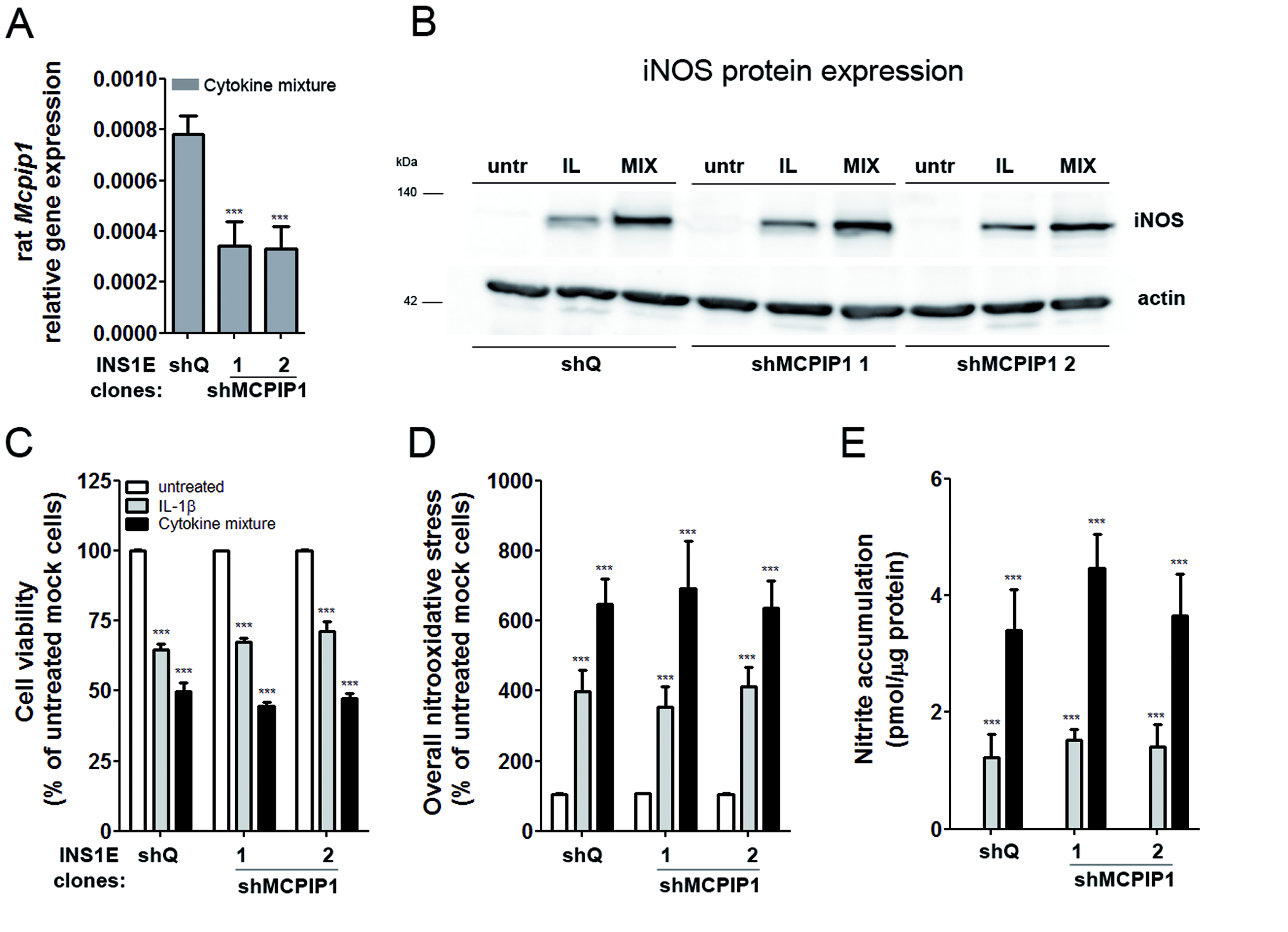
**

**Supplementary Fig. S2**

**
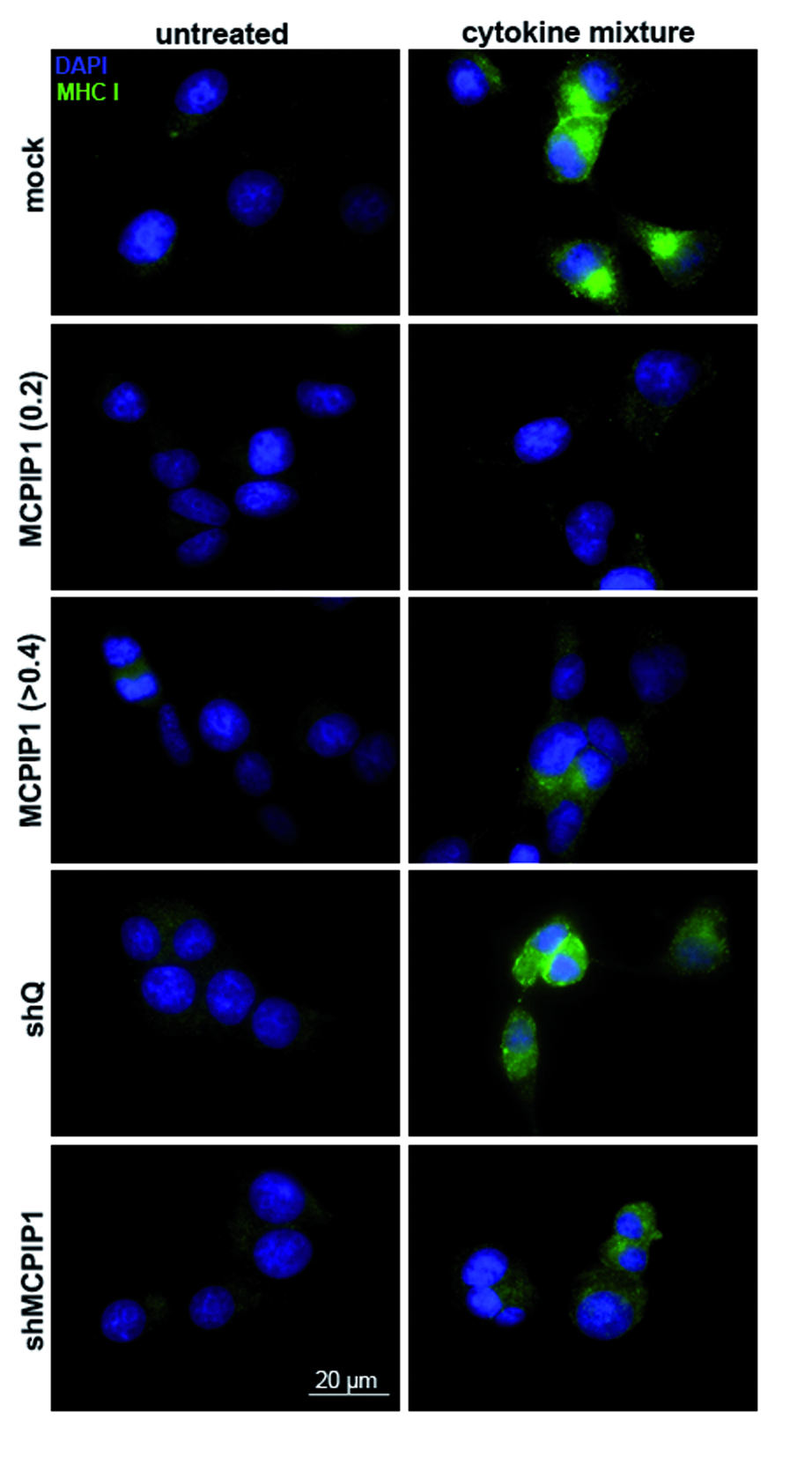
**

**Supplementary Fig. S3**
